# Combat lifesaver-trained, first-responder application of junctional tourniquets: a prospective, randomized, crossover trial

**DOI:** 10.1186/s40779-018-0178-1

**Published:** 2018-09-13

**Authors:** Ismael Flecha, Jason F. Naylor, Steven G. Schauer, Ryan A. Curtis, Cord W. Cunningham

**Affiliations:** 1 21st Combat Support Hospital, Fort Hood, Killeen, 76544 TX USA; 2 Madigan Army Medical Center, JBLM Fort Lewis, Lakewood, 98431 USA; 3 San Antonio Military Medical Center, JBSA Fort Sam Houston, San Antonio, 78234 TX USA; 4 US Army Institute of Surgical Research, JBSA Fort Sam Houston, San Antonio, 78234 TX USA; 5 59th Medical Wing, JBSA Lackland, San Antonio, 78234 TX USA

**Keywords:** Junctional, Tourniquet, Hemorrhage, Combat, Battlefield

## Abstract

**Background:**

Junctional hemorrhage surpassed extremity hemorrhage as the leading cause of preventable death after the resurgence of limb tourniquets during the recent conflicts in Afghanistan and Iraq. Junctional tourniquets (JTQs) were developed in response to this injury pattern. Published data for JTQ efficacy are limited and do not incorporate nonmedical, military first responders. We compared the time for effective placement and scores for device satisfaction between two different JTQs, stratified by combat lifesaver (CLS) and combat medics.

**Methods:**

We performed a prospective, randomized, crossover trial utilizing the SAM® Medical Junctional Tourniquet (SJT) and Junctional Emergency Treatment Tool (JETT™). Investigators simple randomized CLS and combat medics to SJT or JETT for their first JTQ application on mannequins with penetrating inguinal injuries. Then, participants immediately placed the other JTQ on another casualty with the same injury. The primary outcome measured was time of successful application. Success was defined as proper JTQ placement and a pressure reading of at least 180 mmHg. We compared outcomes between CLS and combat medics. Unsuccessful JTQ applications were excluded from the comparative analysis.

**Results:**

From June 2015 to August 2015, a total of 227 personnel (133 CLS and 94 combat medics) at Fort Hood, Texas, USA volunteered to participate in the study. Twenty-eight percent (38 of 133) of CLS and 40% (38 of 94) of combat medics placed both JTQs successfully, for a total of 152 applications (76 SJTs and 76 JETTs). We found a significant difference between applications of the JETT between the CLS and combat medics (92.0 ± 37.7 s versus 70.5 ± 20.5 s, *P =* 0.004). No other subgroup analyses, whether by device or user, demonstrated a significant difference in application time. Both groups preferred the SJT over the JETT. CLS disagreed with combat medics that the JETT could be easily applied by one person (median 3.0 [2.0, 4.0] versus median 4.0 [3.0, 5.0]; *P =* 0.006).

**Conclusion:**

Overall, success rates for both the SJT and JETT were low. Improved training is needed to increase successful application of junctional tourniquets before widespread implementation. Combat lifesavers and combat medics prefer the SJT over the JETT.

## Background

Hemorrhage was the primary source of mortality during the recent conflicts in Afghanistan and Iraq [1, 2]. Junctional hemorrhage surpassed extremity hemorrhage as the leading cause of death from potentially survivable injuries after the resurgence of limb tourniquets [3–6]. In response, multiple devices were developed to control hemorrhage from injuries at the junction of the torso and limbs, but not amenable to limb tourniquet application (e.g., groin and axilla) [7, 8].

As of June 2018, four devices are approved by the US Food and Drug Administration (FDA) for junctional hemorrhage: The Abdominal Aortic and Junctional Tourniquet (AAJT, Compression Works, Birmingham, Alabama, US), the Combat Ready Clamp (CRoC, Combat Medical Systems, Fayetteville, North Carolina, US), the Junctional Emergency Treatment Tool (JETT, North American Rescue, Greer, South Carolina, US), and the SAM Junctional Tourniquet (SJT, SAM Medical Products, Portland, Oregon, US). The AAJT is the only device with a contraindication, since it exerts its compressive forces onto the abdomen [4]. The CRoC is effective, but feedback from military prehospital providers indicates that it is too bulky [9]. The JETT and SJT are the least expensive [9]. The SJT is the only junctional tourniquet (JTQ) that is FDA approved for pelvic stabilization [4].

Pelvic fractures and bilateral leg amputations frequently occur with dismounted improvised explosive device injuries [10]. The selection of the SJT by the US Army Medical Material Agency for inclusion in the Role 1 (point-of-injury to the battalion aid station) medical equipment set seems to address both of these issues [11]. The JETT is the other JTQ that allows bilateral inguinal hemorrhage control common in dismounted complex blast injuries (DCBIs), making the comparison between the SJT and JETT and the exclusion of the AAJT and CRoC reasonable [12]. The US Army has not made the basis of issue plan for the SJT part of the combat lifesaver (CLS) equipment yet, likely due to cost and concern for the ability of CLS to apply the devices.

Previously, published data, using animal, cadaver, and mannequin models, show success with all four devices controlling hemorrhage at the inguinal region [13–19]. Limited studies on live human models demonstrated that all four devices are effective for inguinal injury [20–23]. Published data for JTQ utilization in the military prehospital setting is limited. To date, only a few case reports involving combat casualties have demonstrated variable success [9, 24–26]. Furthermore, previous prospective studies involved only physicians, special operations medics, or senior enlisted combat medics placing the devices [20–22]. It remains unclear whether this technology can be applied by nonmedical, CLS-trained first responders. The goal of this study is to compare time for effective placement of two different JTQ models by CLS-trained first responders versus combat medics. Second, we sought to compare user device preferences.

## Methods

### Study oversight and design

The Brooke Army Medical Center institutional review board approved this prospective, randomized, crossover trial (protocol C.2015.068e). Participant consent was obtained.

### Subjects and materials

Maneuver unit combat medics and CLS conducting annual medical refresher training at the Fort Hood, Texas medical simulation training center (MSTC) were eligible to participate in our study. We had no exclusion criteria. CLS possess no medical qualifications; instead, they are US Army service members of all occupational specialties who volunteer for a week long, basic medical skills training course [27, 28]. Comparatively, combat medics undergo 4 months of advanced individual training as well as continuing medical education at the unit-level and biennial recertification of emergency medical technician [29].

Participants tested on a standardized, combat trauma, simulation lane maintained by the Fort Hood MSTC responsible for delivering realistic medical training [30]. Trainees typically take 40–60 min to complete the outdoor lane that negotiates multiple obstacles, including inoperable vehicles and a helicopter while facility instructors deploy smoke generators and gunfire simulators. Participants wore their military uniform, helmet, ballistic vest, water source, medical treatment bag, and rifle facsimile while negotiating the lane during daylight hours. Simulated casualties consisted of Multiple Amputation Trauma Trainer (MATT) Series 1500 (National Supply Number 6910–01–633-7432; Kforce Government Solutions, Inc., Fairfax, Virginia, US). Mannequins were clothed in military uniforms and possessed a single, identical injury to the right inguinal region simulated with moulage.

Investigators employed Mabis aneroid sphygmomanometers (item code MBS09–141-011; Mabis Healthcare, Inc., Lake Forest, IL, US) to measure JTQ application pressure. We modified a technique originally described by Guirguis and improved by other researchers assessing tourniquet pressures [31–33]. Specifically, we utilized an adult blood pressure cuff, filled it with 60 ml of air, folded it into a 12 cm × 12 cm square, and secured it to the injury site with tape. Investigators instructed participants to stop fastening the JTQ once the pressure reading equaled or surpassed 180 mmHg, which exceeds pressures shown in previous studies to reliably occlude the external iliac artery and common femoral artery [19, 23]. We utilized the SJT (National Stock Number [NSN] 6515–01–618-7475) and JETT (NSN 6515–01–616-5841) devices.

### Study protocol

Participants were consented prior to enrollment. A single investigator (IF) provided all participants with a 20-min block of instruction on SJT and JETT employment, which included hands-on application of both devices. Investigators incorporated simulated casualties with identical injuries and fixed-pressure reading devices into the culminating exercise. Both patients lay next to one another at the end of the trauma lane, with an SJT next to one mannequin and a JETT by the other. Investigators randomized participants to the JTQ utilized first by coin toss. If the first JTQ application was successful, participants moved to the other casualty and placed the other JTQ. If both JTQ applications were successful, then the participant’s attempts were included for comparative analysis. One investigator both evaluated and timed each participant’s attempt. Participants who successfully applied both JTQs completed a survey questionnaire after lane completion in a classroom setting.

### Outcomes

The primary outcome measured was time of successful application in seconds. Investigators started time when the participant touched the device and stopped time when either the application was successful or the time limit of 240 s (4 min) elapsed. Success was defined as proper JTQ placement and a pressure reading of at least 180 mmHg. A time limit of 240 s was based on mean time of successful SJT application reported in previous studies with an additional minute to account for nonmedically trained participants [20, 21]. This time limit correlates with class III hemorrhagic shock and was considered an appropriate benchmark [34]. Secondary outcomes included participant device preference and confidence assessed with a questionnaire utilizing 1–5 Likert items. Questions on the survey were modeled after questions utilized in surveys for previous studies on JTQ [20, 22]. We compared outcomes between CLS and combat medics.

### Statistical analysis

We performed all statistical analyses using Microsoft Excel (version 10, Redmond, Washington, US) and JMP Statistical Discovery from SAS (version 13, Cary, North Carolina, US). We compared study variables using Student’s *t*-test for continuous variables expressed as the means with standard deviations, the Wilcoxon rank sum test for ordinal variables expressed as medians and interquartile ranges, and one-way analysis of variance (ANOVA) for pairwise comparisons. Significance was set as *P* value < 0.05. The power analysis estimated a total of 140 exposures were required to detect a 0.8 standard deviation effect size.

## Results

From June 2015 to August 2015, a total of 227 personnel (133 CLS and 94 combat medics) at Fort Hood, Texas, USA volunteered to participate in the study. Twenty-eight percent (38 of 133) of CLS and 40% (38 of 94) of combat medics placed both JTQ successfully, for a total of 152 applications (76 SJT and 76 JETT). All failed attempts resulted from the participants’ inability to achieve 180 mmHg of tourniquet pressure within 240 s.

We found a significant difference in time for JETT application between CLS and combat medics (92.0 ± 37.7 s versus 70.5 ± 20.5 s, *P =* 0.004, Table 1). No other subgroup analyses, whether by device or user, demonstrated a significant difference in application time. Additionally, the order in which devices were applied secondary to randomization did not result in significant differences in time for successful application (*P =* 0.841).

**Table 1:** Time for junctional tourniquet application

Groups	*n*	Time (s)	95% Confidence interval (s)		*P* value
			Low	High	
All participants					
SJT	76	81.4 ± 35.8	73.1	89.5	0.99
JETT	76	81.3 ± 33.6	73.6	88.9	
Combat Medics					
SJT	38	78.1 ± 36.4	66.1	90.1	0.29
JETT	38	70.5 ± 25.0	62.3	78.7	
Combat Lifesavers					
SJT	38	84.6 ± 35.3	73.0	96.2	0.37
JETT	38	92.0 ± 37.7	79.6	104.4	
SJT					
Combat Medics	38	78.1 ± 36.4	66.1	90.1	0.43
Combat Lifesavers	38	84.6 ± 35.3	73.0	96.2	
JETT					
Combat Medics	38	70.5 ± 25.0	62.3	78.7	0.004^*^
Combat Lifesavers	38	92.0 ± 37.7	79.6	104.4	

*JETT* Junctional Emergency Treatment Tool, *SJT* SAM Junctional Tourniquet; *P value < 0.05

Survey responses indicate CLS and combat medics preferred the SJT over the JETT (Table 2). CLS disagreed with combat medics that the JETT could be easily applied by one person (median 3.0 [2.0, 4.0] versus median 4.0 [3.0, 5.0], *P =* 0.006). Both groups, however, agreed that the SJT could be easily applied by one person. Both groups also indicated that their unit had not implemented training on the JTQ utilized in this study (median 1.0 [1.0, 3.0]).

**Table 2:** Survey results by subgroups

Questions	All Participants	Combat Medics	Combat Lifesavers	Medics vs CLS *P* value
	Median [IQR]	Median [IQR]	Median [IQR]	
I prefer using the JETT because it is easy to adjust compared with the SJT.	2.5 [1.0, 4.0]	3.0 [2.0, 5.0]	2.0 [1.0, 4.0]	0.18
I prefer using the SJT because it is easy to adjust compared with the JETT.	4.0 [2.0, 4.5]	4.0 [2.0, 5.0]	4.0 [3.0, 4.0]	0.23
The application of the SJT can be easily handled by one person.	4.5 [4.0, 5.0]	5.0 [4.0, 5.0]	4.0 [4.0, 5.0]	0.72
The application of the JETT can be easily handled by one person.	4.0 [3.0, 5.0]	4.0 [3.0, 5.0]	3.0 [2.0, 4.0]	0.006^*^

*IQR* Interquartile Range, *JETT* Junctional Emergency Treatment Tool, *SJT* SAM Junctional Tourniquet; *CLS* Combat lifesaver; *P value< 0.05

## Discussion

Overall success rates for JTQ application by both groups of participants were low, with a greater proportion of combat medics successfully applying both JTQ. Among successful interventions, we found that the CLS applied the JETT significantly slower than the combat medics did. Both the CLS and combat medics preferred the SJT over the JETT.

Previously published data demonstrated that JETT applications were slower and less effective than the SJT when applied by physicians and senior combat medics [15, 20–22]. These studies reported success rates of 75%–89% for the JETT and 82%–100% for the SJT [20–22], and also found time for JETT application were 77–212 s, while SJT application time were 65–174 s [20–22]. Although time for successful JTQ application in our study are consistent with the cited reports, the overall success rates for both groups in our study were significantly lower. Unlike the cited reports, however, we incorporated CLS and did not limit combat medic participation to special operations and senior medics only. The experience and training level of our participants may explain the lower success rates we observed. Additionally, our endpoint for successful application was a pressure measurement, while most of the previous studies utilized absence of an arterial pulse by palpation and/ or Doppler transducer. Consequently, methodological differences may have contributed to the variances in outcomes. Lastly, fatigue and more difficult testing conditions inherent to the physically demanding, combat trauma simulation lane may have degraded participant performance. However, these additional challenges are more analogous to the combat setting.

Published reports endorse CLS training of minimally invasive procedures for select Tactical Combat Casualty Care (TCCC) management priorities [35, 36]. Previously published data demonstrate that CLS may successfully place supraglottic airways (SGA), and CLS and nonmedical law enforcement personnel may successfully perform needle chest decompression (NCD) [37–39]. These studies, however, involved limited numbers of participants. The current US Army CLS course trains attendees on NCD but not SGA or JTQ [28]. Future studies evaluating CLS application of JTQ may be beneficial, and should evaluate contributing factors for unsuccessful JTQ applications and minimum training requirements for competency.

Participants of previous studies preferred the SJT over the JETT for JTQ application [15, 20, 22]. We also found that participants preferred the SJT over the JETT, and there was no significant difference in device preference between groups. Additionally, CLS indicated that the JETT is not easily applied by a single person. Our findings are consistent with published data and suggest the SJT may be best suited for CLS JTQ training.

Our study has several important limitations. First, we only compared JTQ applications among participants who successfully placed both devices. We limited data analysis to successful attempts to improve the accuracy of results, but this measure likely introduced selection bias. Second, we are unable to characterize failed attempts or elucidate the potential contributing factors since we did not analyze unsuccessful attempts. Furthermore, we did not retrain and/or retest participants who failed to apply JTQ successfully, so we are unable to provide an estimate of minimum training requirements for CLS and combat medics to achieve acceptable competency. Third, we did not evaluate demographic data or prior experience with JTQs among the participants. Medics or CLS with previous experience with either device may have influenced our findings. However, the majority of participants indicated that their unit did not provide training on either device. Fourth, we utilized a modification of a previously described technique for measuring tourniquet application pressure to determine successful application [31–33]. Although we verified the blood pressure cuff function before and after iterations, we did not calibrate the device for external compressive pressure readings. However, the 180 mmHg pressure that was required for successful application of the device was well above pressures that other studies have previously found to occlude arterial bleeding of the inguinal region [19, 23]. Additionally, our use of a nonbleeding mannequin model and pressure reading as a surrogate for arterial occlusion likely do not closely approximate living tissue physiology. Our results should be viewed as proof-of-concept with future studies validating our findings using other models (e.g., live-tissue, high fidelity mannequins, etc.). Lastly, our study population was localized to one US Army base that may conduct combat medic and CLS training differently than other military installations. Therefore, our findings may not be generalizable to the US military as a whole.

## Conclusion

Overall, success rates for both the SJT and JETT were low. Improved training is needed to increase successful application of junctional tourniquets before widespread implementation. Combat lifesavers and combat medics prefer the SJT over the JETT.

## Abbreviations

AAJT: Abdominal Aortic and Junctional Tourniquet
ANOVA: Analysis of variance
CLS: Combat lifesaver
CRoC: Combat Ready Clamp
DCBI: Dismounted Complex Blast Injuries
FDA: US Food and Drug Administration
JETT: Junctional Emergency Treatment Tool
JTQ: Junctional Tourniquet
MATT: Multiple Amputation Trauma Trainer
MSTC: Medical Simulation Training Center
NCD: Needle chest decompression
NSN: National Stock Number
SGA: Supraglottic airway
SJT: SAM Junctional Tourniquet
TCCC: Tactical Combat Casualty Care
